# BioCompNet: A Deep Learning Workflow Enabling Automated Body Composition Analysis toward Precision Management of Cardiometabolic Disorders

**DOI:** 10.34133/cbsystems.0381

**Published:** 2025-08-20

**Authors:** Jianyong Wei, Hongli Chen, Lijun Yao, Xuhong Hou, Rong Zhang, Liang Shi, Jianqing Sun, Cheng Hu, Xiaoer Wei, Weiping Jia

**Affiliations:** ^1^ Department of Endocrinology and Metabolism, Shanghai Sixth People’s Hospital Affiliated to Shanghai Jiao Tong University School of Medicine, Shanghai Diabetes Institute, Shanghai Key Laboratory of Diabetes Mellitus, Shanghai Key Clinical Center for Metabolic Disease, Shanghai 200233, China.; ^2^ Clinical Research Center, Shanghai Sixth People’s Hospital Affiliated to Shanghai Jiao Tong University School of Medicine, Shanghai 200233, China.; ^3^ Central Research Institute, United Imaging Healthcare, Shanghai 201800, China.; ^4^ Department of Endocrinology and Metabolism, Fengxian Central Hospital Affiliated to Southern Medical University, Shanghai 201499, China.; ^^5^^ Institute of Diagnostic and Interventional Radiology, Shanghai Sixth People’s Hospital Affiliated to Shanghai Jiao Tong University School of Medicine, Shanghai 200233, China.

## Abstract

Growing evidence highlights the importance of body composition (BC), including bone, muscle, and adipose tissue (AT), as a critical biomarker for cardiometabolic risk stratification. However, conventional methods for quantifying BC components using medical images are hindered by labor-intensive workflows and limited anatomical coverage. This study developed BioCompNet—an end-to-end deep learning workflow that integrates dual-parametric magnetic resonance imaging (MRI) sequences (water/fat) with a hierarchical U-Net architecture to enable fully automated quantification of 15 biomechanically critical BC components. BioCompNet targets 10 abdominal compartments (vertebral bone, psoas muscles, core muscles, subcutaneous AT [SAT], superficial SAT, deep SAT, intraperitoneal AT, retroperitoneal AT, visceral AT, and intermuscular AT [IMAT]) and 5 thigh compartments (femur, muscle, SAT, IMAT, and vessels). The workflow was developed on 8,048 MRI slices from a community-based cohort (*n* = 503) and independently validated on 240 MRI slices from a tertiary hospital (*n* = 30). The model’s performance was benchmarked against expert annotations. On internal and external validation datasets, BioCompNet achieved average Dice similarity coefficients of 0.944 and 0.938 for abdominal compartments and 0.961 and 0.936 for thigh compartments, respectively. Excellent interreader reliability was observed (intraclass correlation coefficient ≥ 0.881) across all quantified features, and IMAT quantification showed a strong linear trend (*P*_trend_ < 0.001) compared to physician-rated assessments. The workflow substantially reduced processing time from 128.8 ± 5.6 to 0.12 ± 0.001 min per case. By enabling rapid, accurate, and comprehensive volumetric analysis of BC components, BioCompNet establishes a scalable framework for precision cardiometabolic risk assessment and clinical decision support.

## Introduction

Cardiometabolic diseases (CMDs), including cardiovascular diseases and type 2 diabetes, are leading global causes of morbidity and mortality, posing substantial burdens on individuals and healthcare systems alike [1,2]. Early identification of risk indicators and potential therapeutic targets can help prevent or reduce the loss of lives caused by CMDs [3]. Body composition (BC), comprising adipose tissue, muscle, and bone, has recently emerged as a key biomarker for CMD management [4,5]. Specific BC components, such as visceral adipose tissue (VAT) and subcutaneous adipose tissue (SAT), have demonstrated strong associations with the risk of CMDs [4,6]. Moreover, some of the cytokines secreted by BC components, such as adiponectin, irisin, and osteocalcin, have been shown to be effective early biomarkers of CMDs and have been further developed as drug targets [7]. Despite these advances, the full clinical utility of BC component analysis remains underexplored, largely due to the lack of fast, accurate, and scalable clinical tools for precise quantification in large-scale population-level studies.

Radiological imaging, particularly magnetic resonance imaging (MRI) and computed tomography (CT), is considered the gold standard for BC component assessment and is gradually being employed in large-scale cohort studies [8,9]. However, detailed volumetric quantification requires accurate 3-dimensional (3D) segmentation of individual BC components, a task that is time-consuming and impractical when performed manually by human experts on a slice-by-slice basis [10,11]. There is a pressing need for a reliable, automated solution capable of analyzing 3D BC components with high precision across both community-based populations and clinical cohorts, despite variations in image appearance and pathology.

Automated interpretation of quantitative BC has become a growing field of interest to aid in proactively investigating and managing populations at risk or already affected by CMDs. While earlier traditional automated approaches, such as clustering, thresholding, and active contours techniques, have been applied to segment adipose and muscle tissue, these methods are often constrained by binary tissue classification, reliance on 2-dimensional (2D) data, and limited generalization [12–14]. Recently, deep learning (DL)-based methods have emerged as state-of-the-art in medical image analysis, offering powerful capabilities for high-level feature extraction and pattern recognition [15]. Convolutional neural networks, in particular, have proven highly effective due to their hierarchical structure and ability to capture semantic features [16,17]. Nevertheless, most DL-based BC segmentation studies to date have focused on CT images, which involve radiation exposure and are often limited to abdominal regions [18–20]. MRI-based approaches, while safer and more versatile, have primarily concentrated on specific disease cohorts in hospitals and have not yet achieved comprehensive anatomical coverage [21–23]. For example, Huysmans et al. [24] applied DL-based models to proximal leg muscles from knee to hip in Dixon MRI images in both healthy subjects and patients with muscular dystrophies. However, there is limited research that has simultaneously analyzed multiple BC components across distinct body regions or explored interrelationships among them. Despite the promising clinical implications of using DL to derive composite BC-based biomarkers, a fully automated workflow that can be used to accurately assess the whole-body BC in community populations remains a research gap [25,26].

This study aimed to develop BioCompNet, a convolutional-neural-network-based end-to-end workflow for the rapid and precise quantification of BC, encompassing adipose tissue, muscle, bone, and vessels using abdominal and thigh MRI scans. Compared to existing DL-based BC analysis frameworks, our work introduces the following key advancements:

• Comprehensive anatomical coverage: We provided the first fully automated analysis of 15 distinct BC components across the abdomen and thighs, achieving superior performance compared to prior DL-based methods.
• Dual-channel architecture: Water and fat MRI sequences were integrated to enhance tissue contrast and segmentation accuracy. A 2D U-Net framework was employed to mitigate the impact of MRI data’s anisotropy in 3D space.
• Generalizability and clinical consistency: The proposed model was validated across both community-based and clinical patient cohorts, demonstrating high consistency with physicians’ evaluation and robust cross-population performance.
• Clinical utility and scalability: BioCompNet significantly reduced processing time, offering a scalable DL-based solution for large cohort imaging studies and routine clinical use without increasing physicians’ workload.

## Materials and Methods

### Dataset

**Fig. 1.**
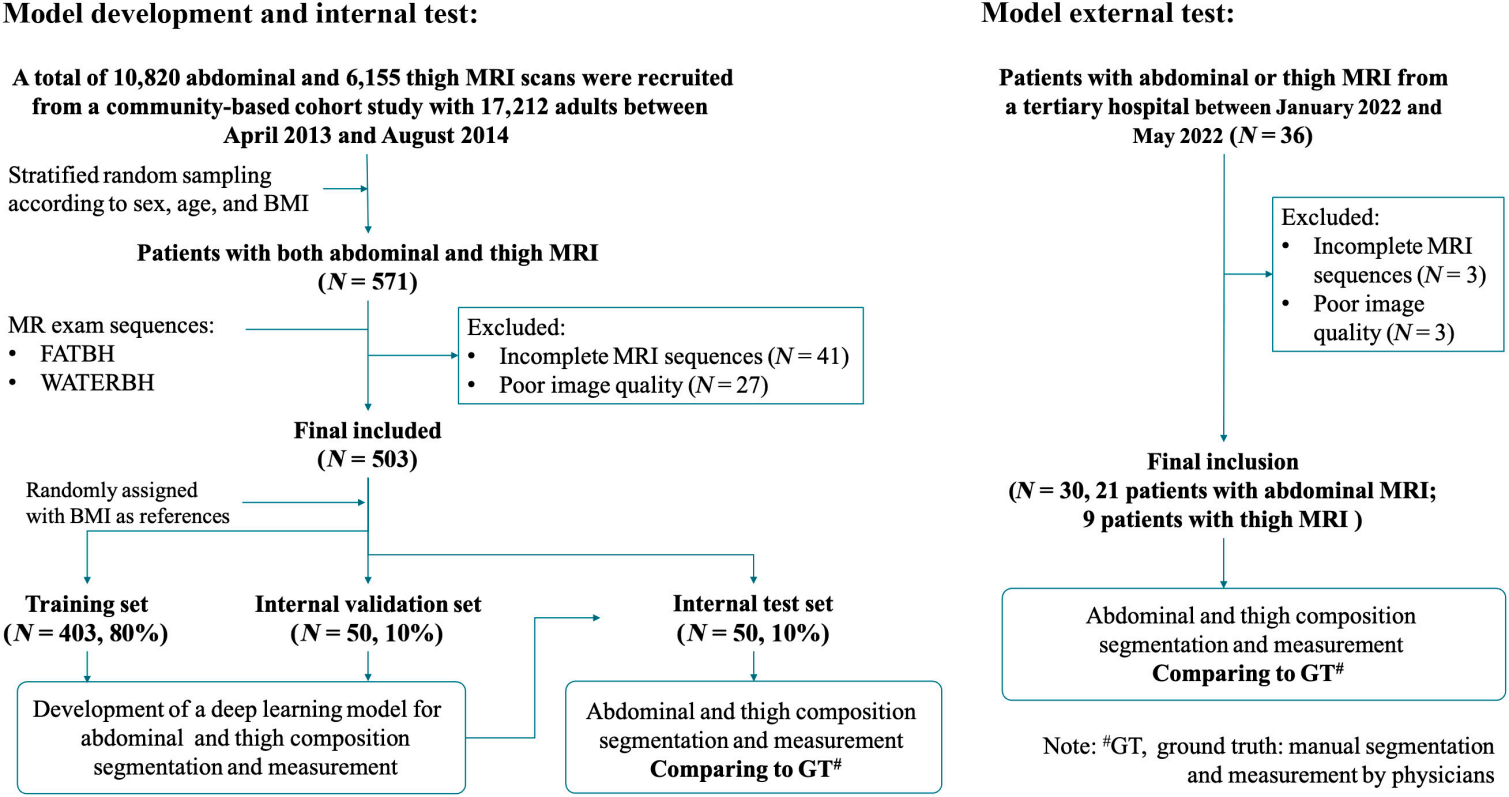
Inclusion and exclusion criteria for model development, internal test, and external test datasets. BMI, body mass index; MRI, magnetic resonance imaging; FATBH, fat breath-hold; WATERBH, water breath-hold.

The model development and testing were based on data from a large-scale, private, community-based prospective cohort study designed to investigate the prevalence, incidence, and associated factors of CMDs. This community-based study recruited a total of 17,212 adults, aged 45 to 70 years, between April 2013 and August 2014. Comprehensive data, such as demographic information, physical examination results, blood and urine samples, and MRI images of the abdomen and thighs, were systematically collected [6]. From this cohort, 571 participants were selected for model development in this study through stratified random sampling based on sex, age, and body mass index (BMI), which were reported to be the major factors affecting BC distribution. After excluding participants with incomplete MRI sequences (*N* = 41) and poor imaging quality due to severe artifacts (*N* = 27), a total of 503 participants (88.1%) were ultimately included (Fig. 1). The final dataset was randomly divided into model training, internal validation, and independent internal test sets at a ratio of 8:1:1, using participants’ BMI as a reference.

Another 36 patients who had undergone abdominal and/or thigh MRI were prospectively enrolled from the outpatient clinic of a tertiary hospital (Shanghai Sixth People’s Hospital) between January 2022 and May 2022 as the independent external test set (Fig. 1). Of these, 30 patients (21 with abdominal MRI and 9 with thigh MRI) were included, after excluding those with incomplete MRI sequences (*N* = 3) and poor imaging quality due to severe artifacts (*N* = 3). The study protocol was approved by the local Ethics Committee of the University Medical Center (Approval No: 2018-010). All study participants provided written informed consent prior to data collection.

### MRI protocol

In the model development and test dataset, all participants underwent abdominal and thigh MRI examinations using a 3.0-T General Electric scanner (GE Healthcare, Milwaukee, WI, USA) equipped with a body coil. Each participant was positioned supine in the magnet and was scanned in cross-sectional planes. T1 axial images were acquired with the abdomen centered on the umbilicus and the thigh centered on the midpoint of the femur, with a slice thickness of 10.0 mm. In the external test dataset, imaging was performed using 2 different MR scanners, with slice thicknesses ranging from 3 to 6 mm. Two imaging sequences, including water-phase and fat-phase images, were acquired for each participant. Detailed scanning parameters are provided in Table S1.

### Ground truth annotation

Eleven anatomical BC components were defined for segmentation (Fig. S1). Among these, the 7 abdominal labels included the vertebral bone, psoas muscles, core muscles (CMs), superficial SAT, deep SAT, intraperitoneal adipose tissue (IPAT), and retroperitoneal adipose tissue (RPAT). The remaining 4 labels, namely, femur, vessel, SAT, and muscle, were located in the thigh region. Manual segmentation and refinement were performed using the 3D Slicer image computing platform (version 5.1.0). Ground truth (GT) annotations were conducted by 2 physicians (H.C. and L.Y., both with 5 years of experience) and supervised by an expert (X.W., with over 10 years of experience in medical imaging) in all datasets. Segmentations were carried out on axial fat-sequence MRI images, which offer superior sensitivity for adipose tissue visualization. Corresponding water-sequence images were also referenced during the annotation process to improve the delineation of anatomical boundaries. To accelerate the annotation while maintaining label quality, an iterative model-assisted annotation strategy was adopted. Initially, 10 cases were manually segmented to train a preliminary 2D U-Net model. Physicians then manually corrected its predictions, and the refined results were used to retrain the model iteratively. Model retraining was conducted iteratively after annotation of 10, 50, 150, and 293 cases, respectively (Fig. S2), with each iteration leveraging the updated annotations to train a new model from scratch. This semiautomatic refinement strategy was employed only during the initial annotation phase to reduce manual workload. Throughout the annotation process, data were randomly divided into training and validation sets in a 7:3 ratio. Finally, all 503 MR examinations were manually reviewed and corrected to ensure label accuracy and consistency.

### Model development

#### Dataset split

The dataset was randomly split into the training set (80%; 403 of 503 subjects), validation set (10%; 50 of 503 subjects), and test set (10%; 50 of 503 subjects), with participants’ BMI used as a stratification reference. An additional external independent test set was used to further evaluate the segmentation model, consisting of 21 subjects with abdominal MRI and 9 subjects with thigh MRI.

#### Data preprocessing

Prior to training and inference, the 3D water–fat MRI sequences of the abdominal and thigh regions were independently normalized using Z-scoring normalization. To address voxel spacing heterogeneity, all MRI images and corresponding annotated masks were resampled to a target spacing of (0.820, 0.820) mm and cropped to an in-plane size of (512, 512) pixels in the axial plane. Third-order spline interpolation was employed for image resampling, while nearest-neighbor interpolation was used for segmentation masks to preserve label integrity. The data augmentation module was stochastically applied to preprocessed images according to a predefined probability of 0.5 during training. The transformations included rotation, scaling, Gaussian noise, Gaussian blur, gamma correction, and mirroring [27]. All augmentation procedures were implemented using the batchgenerators package (https://pypi.org/project/batchgenerators), with parameters consistent with those used in nnU-Net [27].

#### Model architecture

**Fig. 2.**
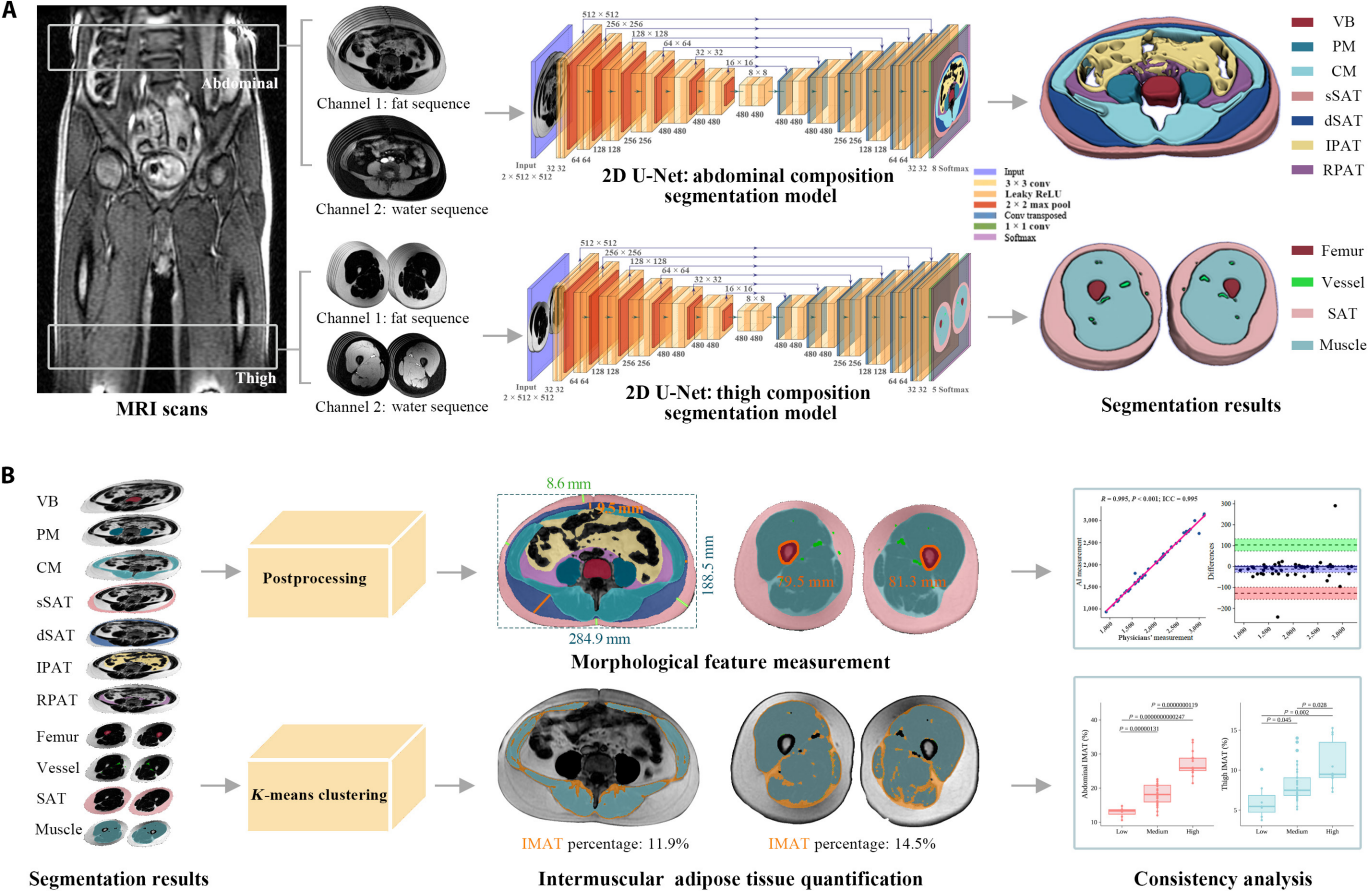
Architectural overview of BioCompNet: a dual-channel deep learning framework for automated body composition analysis from fat-water MRI sequences. (A) Schematic of the dual-channel 2-dimensional (2D) U-Net architecture used to automatically segment abdominal and thigh body composition components from MRI scans. Channel 1 inputs fat-sequence images, which provide enhanced contrast for visualizing adipose tissue, while Channel 2 inputs water-sequence images, which offer better visualization of muscles, bones, and vessels. The network structure is illustrated on the right side using color-coded blocks. The segmented body composition components are represented by different colors for visual differentiation. (B) Based on the segmentation outputs, a fully automated postprocessing algorithm was developed to quantify morphological features (e.g., volume, circumference, and cross-sectional area) for each component. In addition, a *K*-means clustering algorithm (*k* = 2) was applied to the core muscle (CM) and thigh muscle regions to identify intermuscular adipose tissue (IMAT). To assess the accuracy of the automated results produced by BioCompNet, a consistency analysis was performed against reference evaluations conducted by experienced physicians. AI, artificial intelligence; GT, ground truth; MRI, magnetic resonance imaging; VB, vertebral bone; PM, psoas muscle; SAT, subcutaneous adipose tissue; sSAT, superficial subcutaneous adipose tissue; dSAT, deep subcutaneous adipose tissue; IPAT, intraperitoneal adipose tissue; RPAT, retroperitoneal adipose tissue; ICC, intraclass correlation coefficient.

The workflow of the proposed BioCompNet is shown in Fig. 2A. The backbone network, used for segmenting abdominal and thigh BC components, was based on a 2D U-Net architecture [28]. The 2D architecture was strategically chosen to accommodate anisotropic MRI data (e.g., slice thickness 3 to 10 mm vs. in-plane resolution 0.3 to 1.0 mm), avoiding interpolation artifacts inherent in 3D processing [29]. Therefore, the input image contained 2 channels: fat- and water-MRI sequences. The proposed model consists of 6 encoder and 6 decoder convolution blocks, symmetrically connected through a bottleneck block. The architecture processed input images of size 512 × 512 voxels, down-sampling them to 8 × 8 voxel feature maps and subsequently up-sampling them to generate a segmentation mask at the original resolution (512 × 512 voxels). Each encoder block consisted of a convolution block followed by a 2 × 2 max pooling layer for spatial down-sampling. Each decoder block consisted of a 2 × 2 transposed convolution layer for up-sampling followed by a convolution block. Each convolutional block consisted of 2 consecutive 3 × 3 convolution layers padded by 1 × 1 and was followed by a Leaky ReLU activation function with a negative slope of 0.01 [30]. The bottleneck block consisted of a single convolution block with a filter number of (480, 480). The number of filters in the encoder layers was (32, 32), (64, 64), (128, 128), (256, 256), (480, 480), and (480, 480); the decoder layers used the same configuration in reverse order. The final output layer was a 1 × 1 convolution layer with 7 output channels for the abdominal segmentation model and 5 output channels for the thigh segmentation model, respectively. Except for the output layer, both models share the same architecture.

#### Training schedule

All networks were trained for 200 epochs with a batch size of 10, and each epoch comprised 250 minibatches. He et al.’s initialization method [31] was used to initialize the weights of the network. Network weights were optimized using stochastic gradient descent with Nesterov momentum (*μ* = 0.99) and an initial learning rate of 0.01, decayed according to the poly learning rate policy [32]. To enhance the training stability and segmentation accuracy, a composite loss function combining soft Dice loss and cross-entropy loss was employed. The deep supervision method [27] was adopted to facilitate convergence and improve performance across multiple network layers. All procedures were implemented in Python (version 3.11) and PyTorch (version 2.5.1) libraries and executed on a Linux server equipped with an NVIDIA GeForce RTX 3090 graphics processing unit.

#### Model segmentation evaluation metrics

The performance of the segmentation model was quantitatively evaluated using the following metrics. These metrics were chosen to comprehensively assess the overlap accuracy, sensitivity, and volumetric agreement between the predicted segmentation and the GT annotations.

• Dice similarity coefficient (DSC)

$$\text{DSC}=\frac{2\times \text{TP}}{2\times \text{TP}+\text{FP}+\text{FN}}$$ (1)

The DSC measures the spatial overlap between the predicted and GT segmentations. It ranges from 0 to 1, where 1 indicates perfect overlap. This metric is widely used in medical image segmentation due to its robustness in evaluating overlap.

• Jaccard score

$$\text{Jaccard score}=\frac{\text{TP}}{\text{TP}+\text{FP}+\text{FN}}$$ (2)

This metric assesses the ratio between the intersection and union of the predicted and GT segmentations. It is a more stringent measure than DSC and also ranges from 0 to 1, with higher values indicating better segmentation performance.

• True-positive fraction (TPF)

$$\text{TPF}=\frac{\text{TP}}{\text{TP}+\text{FN}}$$ (3)

Also known as sensitivity or recall, TPF measures the proportion of actual positive pixels correctly identified by the model. The range is 0 to 1, with 1 indicating perfect sensitivity.

• Oversegmentation rate (OSR)

$$\text{OSR=}\frac{\mathrm{FP}}{\mathrm{TP}+\mathrm{FN}}$$ (4)

This metric quantifies the proportion of incorrectly predicted positive pixels relative to all actual positive pixels, indicating oversegmentation. Lower values are preferred, with 0 representing ideal performance.

• Area difference (%)

$$\text{Area difference}=\frac{\left|\left(\text{TP}+\text{FP}\right)-\left(\mathrm{TP}+\text{FN}\right)\right|}{\text{TP}+\text{FN}}\times 100$$ (5)

This metric evaluates the relative difference in segmented area (or volume) between the prediction and the GT. Smaller values suggest better agreement in terms of volume quantification.

 TP, FP, FN, and TN refer to the number of true-positive, false-positive, false-negative, and true-negative pixels, respectively. All metrics were calculated on a per-case basis and averaged across test sets to ensure robust evaluation.

### Experiments

#### Ablation study

To evaluate the individual contributions of input modalities and the data augmentation module to segmentation performance, an ablation study was conducted using 4 model variants: fat-only, water-only, dual-sequence without data augmentation, and dual-sequence with data augmentation (BioCompNet). All variants were trained using the same training and internal validation sets. Their segmentation performance was consistently evaluated on both the internal test set and an independent external test set to ensure comparability and robustness.

#### Comparative study with benchmark models

To further evaluate the performance and generalizability of the proposed BioCompNet, we compared it against 2 widely used segmentation benchmark models: the 2D nnU-Net and the 3D full-resolution nnU-Net [27]. Both models were trained using the same preprocessed training dataset and evaluated using the same internal and external test sets as BioCompNet. The nnU-Net implementations were based on the official open-source framework (https://github.com/MIC-DKFZ/nnUNet/tree/master/nnunetv2), with consistent hyperparameters and training procedures across models to ensure a fair comparison. While both BioCompNet and the 2D nnU-Net were trained in a slice-wise manner, the 3D nnU-Net operated on volumetric inputs using anisotropic patch sizes tailored to the spatial resolution of the MRI scans.

### Feature quantification based on DL segmentation

Following the implementation of the proposed DL model for the segmentation of BC components, we further quantified the morphological features and intermuscular adipose tissue (IMAT) in the abdomen and thigh regions using a fully automated postprocessing pipeline (Fig. 2B). Morphological features associated with metabolic disorders [9,33–35], such as the volume of bone, muscle, and adipose tissues, were computed directly from the segmentation mask without any additional morphological refinement. To specifically quantify IMAT within the CM region of the abdominal image and the muscle region in the thigh image, we applied a *K*-means clustering algorithm [36] with 2 clusters (cluster number *k* = 2) to the pixel intensities within the regions of interested on fat-sequence MRI images. The cluster with the higher mean intensity was identified as IMAT. The *K*-means++ initialization strategy was employed to enhance convergence stability. The clustering process was terminated based on a relative tolerance of 1e−4 (measured by the Frobenius norm of changes in cluster centers across iterations) or a maximum of 300 iterations.

To validate the accuracy of the automated measurements, we compared the postprocessing pipeline outputs with a reference standard comprising semiautomatic evaluations performed by 2 experienced physicians (H.C. and L.Y., over 5 years of experience), conducted using the 3D Slicer platform (version 5.1.0). These assessments were performed blinded to other clinical information. Discrepancies were arbitrated by a senior radiologist with over 10 years of experience (X.W.). The physicians’ evaluation of IMAT was graded on a 3-level scale based on the Goutallier classification standard [37]: mild (stage 0, with no fatty infiltration), moderate (stage 1, few fatty streaks within the muscle), and severe (stage 2, less than 50% fat within the muscle) infiltration. The time efficiency of the automated method was also compared with that of manual assessment.

### Statistical analysis

All analyses were conducted using Python (version 3.10.4) and R software (version 4.0.4, R Foundation for Statistical Computing, Vienna, Austria). Continuous variables were reported as mean ± standard deviation (SD) if normally distributed or as median and interquartile range (IQR) otherwise. Categorical variables were summarized as frequencies and percentages. To assess agreement between BioCompNet and physician evaluations in measuring morphological features, we employed the Pearson correlation coefficient, intraclass correlation coefficient (ICC), and Bland–Altman analysis. ICC was calculated using a 2-way random effects model for absolute agreement [38], appropriate when all subjects are evaluated by the same raters (i.e., model and physician) and the interest lies in the absolute values rather than consistency alone. Specifically, ICC was applied to continuous variables such as volumes of segmented BC components. Bland–Altman plots were used to visualize agreement by plotting the differences between model-derived and physician-derived measurements against their means [39]. The 95% limits of agreement were calculated as mean difference ± 1.96 × SD of the differences. To evaluate the linear association between model-predicted IMAT values and ordinal grades assigned by physicians, we applied a linear regression model, treating the ordinal grades as continuous predictors to test for a significant trend [40]. An independent *t* test was used to compare measurements across groups. All statistical tests were 2-sided, with a significance threshold of *P* < 0.05. Statistical analysis was performed by 2 authors (J.W. and H.C.), both with over 6 years of experience in algorithm development.

## Results

### Participants’ characteristics

A total of 8,048 MRI slices (one axial section from each examination) collected from 503 participants (median age [IQR], 57.5 years [52.0 to 62.1]; 181 males) were included in the model development and internal test datasets. For the external test dataset, 21 patients (median age [IQR], 42.0 years [38.0 to 61.0]; 7 males) with abdominal MRI and 9 patients (median age [IQR], 29.0 years [15.0 to 34.0]; 4 males) with thigh MRI were enrolled. The baseline characteristics for each dataset are summarized in Table 1.

**Table 1.** Characteristics of the participants. Note: ^#^*P* value for comparison between the training and validation set and the internal test set. ^*^P value for comparison between the internal test set and the external test set. Skewed variables are presented as medians (interquartile ranges). Categorical variables are presented as frequencies (%). A *P* value of less than 0.05 indicates a statistically significant difference between groups.

Characteristics	Model development set				External test set	P*
	All	Training and validation set	Internal test set	*P* ^#^		
*N*	503	453	50		30	
Age, years	57.5 (52.0–62.1)	57.5 (52.0–62.1)	56.5 (52.5–62.4)	0.879	42.0 (30.3–56.8)	0.021
<50	80 (15.9%)	73 (16.1%)	7 (14.0%)		18 (60.0%)	
50–54	100 (19.9%)	90 (19.9%)	10 (20.0%)		4 (13.3%)	
55–59	152 (30.2%)	139 (30.7%)	13 (26.0%)		1 (3.4%)	
60–64	99 (19.7%)	88 (19.4%)	11 (22.0%)		3 (10.0%)	
≥65	72 (14.3%)	63 (13.9%)	9 (18.0%)		4 (13.3%)	
Male	181 (36.0%)	157 (34.7%)	24 (48.0%)	0.087	11 (36.7%)	0.062
BMI/(kg·m^−2^)	25.2 (23.2–27.4)	25.2 (23.2–27.4)	24.8 (23.4–27.0)	0.637	23.9 (23.1–27.1)	0.546
<18.5	7 (1.4%)	4 (0.9%)	3 (6.0%)	0.059	1(3.3%)	0.056
18.5–23.9	167 (33.3%)	154 (34.1%)	13 (26.0%)		8 (26.7%)	
24.0–27.9	230 (45.8%)	206 (45.5%)	24 (48.0%)		14 (46.7%)	
≥28.0	98 (19.5%)	88 (19.5%)	10 (20.0%)		7 (23.3%)	
NAFLD	266 (52.9%)	241 (53.2%)	25 (50.0%)		ND	
T2DM	126 (25.0%)	115 (25.4%)	11 (22.0%)		ND	
CVD	6 (1.2%)	6 (1.3%)	0 (0.0%)		ND	
CKD	35 (7.0%)	32 (7.1%)	3 (6.0%)		ND	

BMI, body mass index; NAFLD, nonalcoholic fatty liver disease; T2DM, type 2 diabetes mellitus; CVD, cardiovascular disease; CKD, chronic kidney disease

### Experiments and model segmentation performance

#### Ablation study results

The average DSCs for abdominal and thigh segmentation across both the internal and external test sets of the ablation study are summarized in Table 2. The results demonstrated that the dual-sequence model consistently outperformed single-sequence models in both anatomical regions, confirming the benefit of integrating fat- and water-sequence MRI images as input. Notably, incorporating the data augmentation module could significantly improve segmentation performance, particularly on the external test set, underscoring the enhanced generalizability of the model. For instance, in the abdominal region of the external test set, the average DSC increased from 0.907 (without data augmentation) to 0.938 (with data augmentation), while in the thigh region, the DSC improved from 0.928 to 0.936. These findings validated the effectiveness of the proposed data augmentation module in boosting model robustness and mitigating overfitting. Similar trends were observed across all components (detailed in Table S2).

**Table 2.** Average segmentation DSC for the ablation study across different model variants. Note: Data are reported as mean ± standard deviation. The reported values represent the average DSC across all segmented body composition compartments in the abdomen and thigh regions.

Dataset	Fat-only with DA	Water-only with DA	Dual-sequence without DA	Dual-sequence with DA (BioCompNet)
Internal test set				
Abdominal	0.926 ± 0.028	0.931 ± 0.026	0.939 ± 0.023	0.944 ± 0.019
Thigh	0.954 ± 0.018	0.954 ± 0.014	0.940 ± 0.131	0.961 ± 0.015
External test set				
Abdominal	0.905 ± 0.054	0.909 ± 0.058	0.907 ± 0.091	0.938 ± 0.042
Thigh	0.905 ± 0.048	0.902 ± 0.038	0.928 ± 0.017	0.936 ± 0.047

DA, data augmentation; DSC, Dice similarity coefficient

**Fig. 3.**
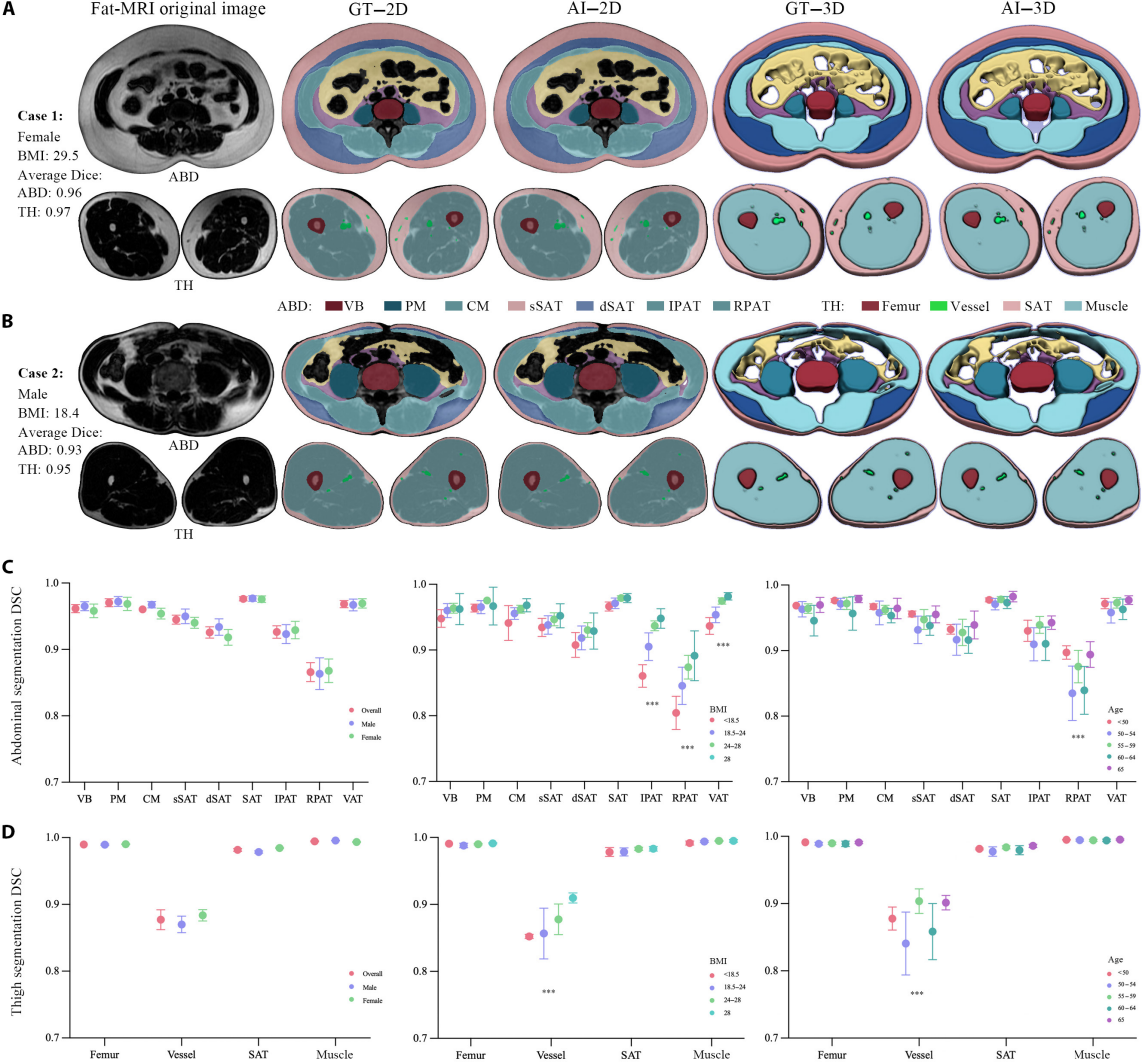
Segmentation performance of the proposed deep learning model on the internal test set. (A) and (B) illustrate 2 representative cases of abdominal and thigh body composition segmentation on MRI images using BioCompNet. The fat-MRI column shows the original axial fat-sequence MRI images, with the upper row corresponding to abdominal slices and the lower row to thigh slices. For each case, the second and fourth columns present ground truth annotations of tissue compartments, visualized in both 2 dimensions and 3 dimensions. The third and fifth columns show the corresponding BioCompNet predictions. Each tissue component is color coded for clarity. (C) and (D) summarize the quantitative segmentation performance using DSCs across 7 abdominal components and 4 thigh components. Bar plots indicate mean DSC values with 95% confidence intervals. The first column shows DSC distributions across the entire dataset as well as subgroups stratified by sex. The second and third columns present results stratified by BMI and age categories, respectively. Multiple subgroup comparisons are conducted using statistical tests; *** indicates statistically significant differences between subgroups (*P* < 0.001). ABD, abdominal; TH, thigh; BMI, body mass index; MRI, magnetic resonance imaging; DSC, Dice similarity coefficient; VB, vertebral bone; PM, psoas muscle; CM, core muscle; SAT, subcutaneous adipose tissue; sSAT, superficial subcutaneous adipose tissue; dSAT, deep subcutaneous adipose tissue; VAT, visceral adipose tissue; IPAT, intraperitoneal adipose tissue; RPAT, retroperitoneal adipose tissue.

The BioCompNet model, incorporating a dual-sequence architecture and a data augmentation module, achieved high accuracy in BC segmentation. The performance metrics of each tissue component on the internal and external test sets are shown in Table 3. Representative 2D and 3D segmentation results generated by BioCompNet are shown in Fig. 3A and B. BioCompNet achieved a segmentation of abdominal and thigh compositions with average DSCs of 0.944 and 0.961 on the internal test set and 0.938 and 0.936 on the external test set, respectively. The DSCs were the highest in the SAT and relatively low in the RPAT. The box plots in Fig. 3C and D illustrate segmentation performance across subgroups stratified by sex and age, revealing no significant differences in DSC across these categories. However, noticeable variations in segmentation DSCs for abdominal VAT, IPAT, and RPAT were observed across BMI- and nonalcoholic-fatty-liver-disease (NAFLD)-stratified subgroups, while segmentation performance for thigh vessels varied across subgroups stratified by both BMI and age (see representative cases in Fig. S3 and disease-stratified subgroup analysis in Fig. S4).

**Table 3.** Segmentation performance of BioCompNet on the internal test and external test datasets. Note: Data are reported as mean ± standard deviation. SAT includes areas of both sSAT and dSAT. VAT includes areas of both IPAT and RPAT.

Compartment	DSC	Jaccard score	True-positive fraction	Oversegmentation rate	Area difference /%
Internal test set					
Abdominal					
VB	0.962 ± 0.021	0.927 ± 0.038	0.962 ± 0.037	0.038 ± 0.036	4.006 ± 4.395
PM	0.970 ± 0.021	0.943 ± 0.037	0.973 ± 0.021	0.032 ± 0.027	1.425 ± 1.746
CM	0.960 ± 0.018	0.924 ± 0.032	0.966 ± 0.017	0.046 ± 0.025	1.832 ± 1.821
SAT	0.976 ± 0.011	0.954 ± 0.020	0.974 ± 0.012	0.022 ± 0.019	1.681 ± 1.659
sSAT	0.945 ± 0.023	0.896 ± 0.041	0.946 ± 0.025	0.057 ± 0.034	2.751 ± 2.336
dSAT	0.926 ± 0.030	0.863 ± 0.051	0.923 ± 0.036	0.071 ± 0.048	4.250 ± 4.113
VAT	0.968 ± 0.019	0.939 ± 0.035	0.968 ± 0.021	0.031 ± 0.021	1.390 ± 1.411
IPAT	0.926 ± 0.033	0.864 ± 0.055	0.916 ± 0.043	0.061 ± 0.041	4.252 ± 4.178
RPAT	0.866 ± 0.049	0.767 ± 0.074	0.896 ± 0.049	0.177 ± 0.106	9.980 ± 9.733
Thigh					
Femur	0.990 ± 0.004	0.980 ± 0.007	0.988 ± 0.007	0.009 ± 0.003	0.549 ± 0.606
Vessel	0.877 ± 0.052	0.785 ± 0.078	0.859 ± 0.083	0.095 ± 0.042	7.254 ± 8.108
SAT	0.982 ± 0.007	0.964 ± 0.014	0.984 ± 0.007	0.021 ± 0.010	0.711 ± 0.594
Muscle	0.994 ± 0.002	0.989 ± 0.004	0.994 ± 0.003	0.006 ± 0.002	0.242 ± 0.279
External test set					
Abdominal					
VB	0.960 ± 0.023	0.924 ± 0.040	0.983 ± 0.018	0.066 ± 0.052	5.616 ± 5.296
PM	0.966 ± 0.047	0.938 ± 0.077	0.964 ± 0.072	0.029 ± 0.022	3.221 ± 5.943
CM	0.968 ± 0.026	0.939 ± 0.044	0.971 ± 0.013	0.037 ± 0.050	2.094 ± 4.081
SAT	0.985 ± 0.008	0.971 ± 0.016	0.987 ± 0.012	0.017 ± 0.006	0.769 ± 0.786
dSAT	0.916 ± 0.056	0.849 ± 0.091	0.953 ± 0.046	0.134 ± 0.122	10.618 ± 11.461
sSAT	0.939 ± 0.031	0.887 ± 0.055	0.920 ± 0.058	0.037 ± 0.027	6.284 ± 5.212
VAT	0.963 ± 0.058	0.934 ± 0.091	0.972 ± 0.030	0.052 ± 0.119	3.288 ± 9.773
IPAT	0.906 ± 0.066	0.835 ± 0.098	0.914 ± 0.079	0.103 ± 0.091	7.169 ± 6.966
RPAT	0.836 ± 0.116	0.732 ± 0.138	0.852 ± 0.066	0.225 ± 0.332	17.585 ± 24.849
Thigh					
Femur	0.960 ± 0.024	0.924 ± 0.044	0.932 ± 0.043	0.009 ± 0.006	6.026 ± 4.077
Vessel	0.841 ± 0.035	0.727 ± 0.052	0.784 ± 0.069	0.078 ± 0.041	14.904 ± 8.098
SAT	0.961 ± 0.012	0.924 ± 0.022	0.963 ± 0.020	0.041 ± 0.013	2.186 ± 0.975
Muscle	0.984 ± 0.004	0.967 ± 0.008	0.989 ± 0.006	0.021 ± 0.006	1.161 ± 0.574

DSC, Dice similarity coefficient; VB, vertebral bone; PM, psoas muscle; CM, core muscle; SAT, subcutaneous adipose tissue; sSAT, superficial subcutaneous adipose tissue; dSAT, deep subcutaneous adipose tissue; VAT, visceral adipose tissue; IPAT, intraperitoneal adipose tissue; RPAT, retroperitoneal adipose tissue

#### Comparative evaluation with benchmark models

The comparative study demonstrated that the proposed 2D BioCompNet achieved segmentation performance comparable or superior to those of both the 2D and 3D nnU-Net across internal and external test sets. Table 4 summarizes the performance of all 3 models across multiple metrics. On the internal test set, all models produced similar results. The 2D nnU-Net slightly outperformed others in abdominal segmentation, achieving an average DSC of 0.947, while BioCompNet matched its performance in thigh segmentation with an average DSC of 0.961. However, on the external test set, BioCompNet outperformed both benchmarks, achieving the highest DSC in abdominal (0.938) and thigh (0.936) segmentation, demonstrating superior robustness to domain shifts. Notably, the 3D nnU-Net showed inferior performance on external data, particularly in the thigh region (DSC: 0.912), indicating a limited ability to generalize across domains. These results supported the effectiveness of our customized 2D BioCompNet in achieving robust and reliable segmentation performance across diverse datasets.

**Table 4.** Quantitative comparison of segmentation performance across different models on internal and external test sets for abdominal and thigh regions. Note: Data are reported as mean ± standard deviation. The reported values represent the average DSC across all segmented body composition compartments in the abdomen and thigh regions.

Method	DSC	Jaccard score	True-positive fraction	Oversegmentation rate	Area difference /%
Internal test set—ABD					
2D nnU-Net	0.947 ± 0.018	0.903 ± 0.032	0.949 ± 0.018	0.057 ± 0.021	3.491 ± 1.864
3D nnU-Net	0.946 ± 0.017	0.899 ± 0.030	0.949 ± 0.016	0.059 ± 0.022	3.716 ± 2.037
BioCompNet (ours)	0.944 ± 0.019	0.898 ± 0.033	0.947 ± 0.018	0.059 ± 0.023	3.508 ± 2.062
Internal test set—TH					
2D nnU-Net	0.961 ± 0.015	0.929 ± 0.024	0.956 ± 0.023	0.031 ± 0.010	2.265 ± 2.214
3D nnU-Net	0.960 ± 0.015	0.927 ± 0.023	0.954 ± 0.023	0.033 ± 0.010	2.402 ± 2.279
BioCompNet (ours)	0.961 ± 0.015	0.929 ± 0.023	0.956 ± 0.022	0.033 ± 0.011	2.190 ± 2.116
External test set—ABD					
2D nnU-Net	0.931 ± 0.044	0.879 ± 0.065	0.939 ± 0.045	0.078 ± 0.044	5.803 ± 5.191
3D nnU-Net	0.919 ± 0.048	0.858 ± 0.069	0.922 ± 0.053	0.085 ± 0.038	6.506 ± 4.901
BioCompNet (ours)	0.938 ± 0.042	0.890 ± 0.061	0.946 ± 0.032	0.078 ± 0.070	6.293 ± 5.870
External test set—TH					
2D nnU-Net	0.927 ± 0.015	0.871 ± 0.023	0.904 ± 0.021	0.040 ± 0.008	7.125 ± 2.381
3D nnU-Net	0.912 ± 0.014	0.849 ± 0.022	0.879 ± 0.018	0.036 ± 0.010	10.99 ± 2.355
BioCompNet (ours)	0.936 ± 0.015	0.886 ± 0.024	0.917 ± 0.023	0.037 ± 0.010	6.069 ± 2.431

ABD, abdomen; TH, thigh; DSC, Dice similarity coefficient

### Accuracy of feature quantification after DL segmentation

**Fig. 4.**
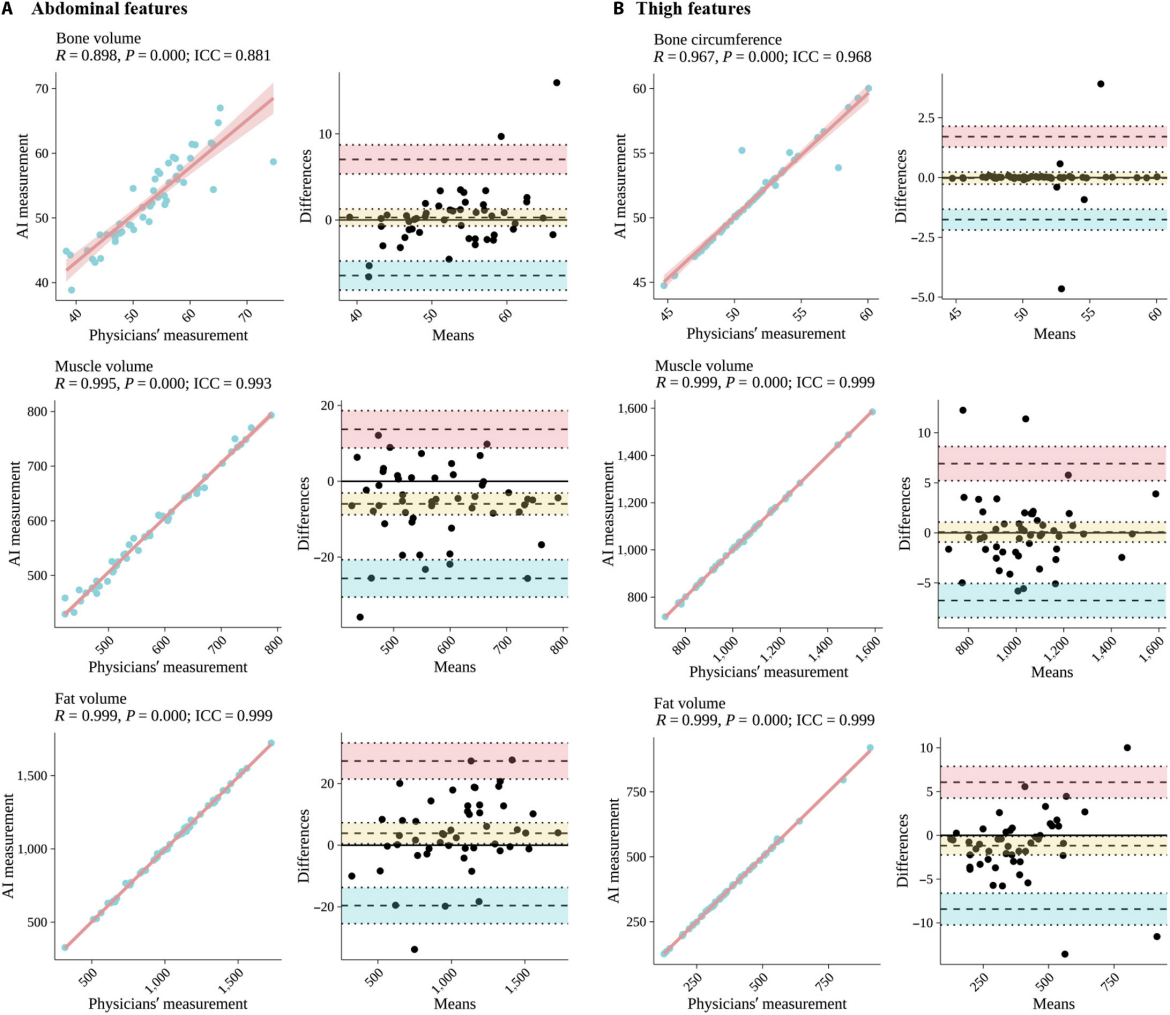
Consistency analysis between BioCompNet and physician-derived measurements for abdominal and thigh body composition components. (A) Linear regression plots (left) and Bland–Altman plots (right) demonstrate agreement between AI-based model (BioCompNet) and physician measurements for abdominal components: bone volume (top), muscle volume (middle), and fat volume (bottom). (B) Linear regression plots (left) and Bland–Altman plots (right) show agreement for thigh components: bone circumference (top), muscle volume (middle), and fat volume (bottom). A *P* value <0.05 indicates statistically significant agreement between the 2 methods. An ICC with a value of 1.0 represents perfect agreement. Each dot represents an individual subject. Solid lines in regression plots denote the line of best fit; dashed lines in Bland–Altman plots indicate mean bias and limits of agreement (±1.96 standard deviations). AI, artificial intelligence; ICC, intraclass correlation coefficient.

**Fig. 5.**
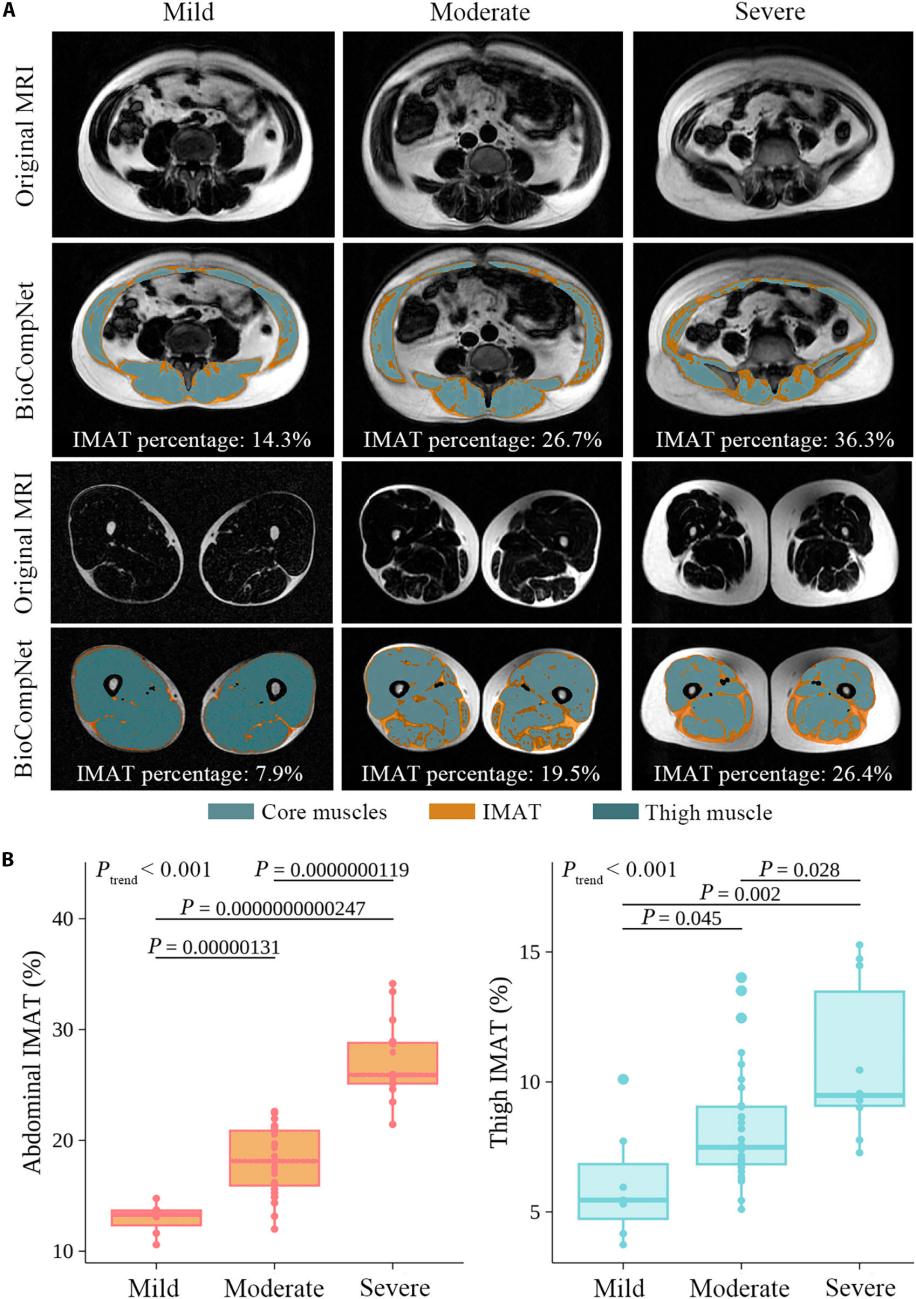
Rater agreement of IMAT. (A) A consensus is reached among experienced physicians who categorized IMAT into 3 levels: mild (first column), moderate (second column), and severe (third column), based on the visual assessment of the original MRI images in the fat sequence. The automatic quantification of IMAT is conducted using the proposed BioCompNet. The IMAT percentage is defined as the volume of IMAT divided by the total muscle volume. (B) Box plots for rater agreement of IMAT between BioCompNet and experienced physicians. Boxes represent interquartile range, and centerlines denote the median. Statistical comparison among groups was performed using independent-sample *t* tests. A trend test was conducted using a linear regression model. *P* < 0.05 indicates statistically significant difference. MRI, magnetic resonance imaging; IMAT, intermuscular adipose tissues.

Among the 50 scans in the internal test dataset, the agreement between BioCompNet and experienced physicians in quantifying the volume of abdominal and thigh bones, muscles, and adipose tissues is illustrated in Fig. 4. The rater examination ICCs for these measurements ranged from 0.881 up to values exceeding 0.999. Bland–Altman plots further demonstrated minimal differences and narrow 95% limits of agreement between the 2 methods, confirming the high accuracy of feature quantification achieved by BioCompNet. Fig. 5A presents examples of BioCompNet-derived IMAT percentages corresponding to mild, moderate, and severe infiltration in both the abdomen and thigh regions, which closely align with manual assessments. The consistency analysis of IMAT in the CM and thigh muscle is summarized in Fig. 5B, demonstrating a strong correlation between the automated IMAT quantification and assessments by experienced physicians’ evaluations (*P*_trend_ < 0.001). The processing time for the entire workflow, encompassing abdominal and thigh segmentations through feature analysis, was only 0.12 ± 0.001 min for BioCompNet, in contrast to 128.8 ± 5.6 min required manually for each case.

### BC distribution of enrolled participants

**Fig. 6.**
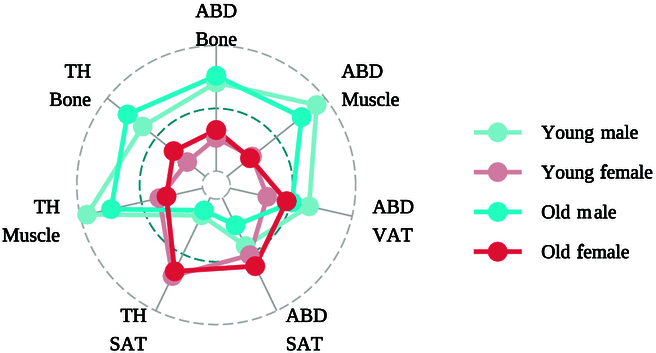
Sex- and age-specific body composition distribution. The radar plot shows the distributions of abdominal and thigh compositions, including volumes of bone, muscle, and fat, stratified by sex and age. The cut-off age of the participants is 50 years. ABD, abdominal; TH, thigh; VAT, visceral adipose tissue; SAT, subcutaneous adipose tissue.

Following segmentation and quantification, the BC metrics were used to characterize the phenotypic profiles of the study population. As illustrated in Fig. 6, radar plots were utilized to depict the distributions of BC components in the abdominal and thigh compositions, stratified by sex and age. The results revealed noticeable differences in BC among various subgroups. In general, the volumes of bone, muscle, and VAT were generally higher in males than in females, whereas abdominal SAT and thigh SAT were lower in males than in females. Additionally, with increasing age, males exhibited a marked decrease in muscle mass, while the BC distribution in female showed a progressive masculinization, as indicated by an increase in abdominal adipose tissue.

### Latest datasets and performance

Existing studies on adipose tissue quantification have primarily employed various image segmentation techniques (e.g., 2D U-Net, 3D DCNet, and DenseNet) using either MRI or CT imaging modalities (as summarized in Table 5) [11,18,19,22–24,41]. A common theme in these studies was the application of DL models to accurately quantify abdominal adipose tissue, including SAT and VAT. Many reported excellent performances, with DSC and ICC values ranging from 0.9 to 0.99, indicating reliable and consistent segmentation results. However, several aspects distinguish these studies. First, while most focused exclusively on abdominal fat, a few extended their analysis to the thigh or femoral regions, although with varying degrees of success. Second, although DL approaches have consistently demonstrated improved accuracy, the specific model architecture (e.g., 2D vs. 3D) and its application can influence performance, especially when targeting multiregion quantification. Third, studies with larger sample sizes generally reported more robust results, although smaller studies could still produce reliable findings. Participants’ diversity in terms of demographics and BC components was also an important factor influencing the generalizability of the results.

**Table 5.** Comparison of the body composition qualification performance of this study with those of previous studies. Note: SAT includes areas of both sSAT and dSAT. VAT includes areas of both IPAT and RPAT.

Study	Method	Participants	Modality	Abdominal composition quantification (^*^DSC, ^#^ICC)									Thigh composition quantification (^*^DSC, ^#^ICC)			
				VB	PM	CM	sSAT	dSAT	SAT	IPAT	RPAT	VAT	Femur	Vessel	SAT	Muscle
2017 Middleton et al. [11]	Semiautomated	20	MRI	ND	ND	ND	ND	ND	0.998^#^	ND	ND	0.998^#^	ND	ND	ND	0.998^#^
2019 Weston et al. [18]	2D U-Net	270	CT	0.98^*^	ND	0.95^*^	ND	ND	0.98^*^	ND	ND	0.99^*^	ND	ND	ND	ND
2020 Estrada et al. [41]	2D CDFNet	641	MRI	ND	ND	ND	ND	ND	0.975^*^	ND	ND	0.850^*^	ND	ND	ND	ND
2020 Kustner et al. [22]	3D DCNet	1,000	MRI	ND	ND	0.91^*^	ND	ND	0.95^*^	ND	ND	0.89^*^	ND	ND	ND	ND
2021 Magudia et al.[47]	DenseNet	604	CT	ND	ND	ND	ND	ND	0.97^*^	ND	ND	0.95^*^	ND	ND	ND	ND
2021 Kway et al. [23]	VGG-16	1,088	MRI	ND	ND	ND	0.943^*^	0.844^*^	ND	ND	ND	0.955^*^	ND	ND	ND	ND
2022 Bridge et al. [19]	U-Net	629	CT	ND	ND	0.985^#^	ND	ND	0.994^#^	ND	ND	0.994^#^	ND	ND	ND	ND
2023 Huysmans et al. [24]	3D U-Net	106	MRI	ND	ND	ND	ND	ND	ND	ND	ND	ND	ND	ND	ND	0.953^*^
Ours	2D U-Net	503	MRI	0.962^*^/0.881^#^	0.970^*^	0.960^*^/0.993^#^	0.945^*^/0.999^#^	0.926^*^/0.999^#^	0.976^*^/0.999^#^	0.926^*^	0.866^*^	0.968^*^/0.999^#^	0.990^*^/0.968^#^	0.877^*^	0.982^*^/0.999^#^	0.994^*^/0.999^#^

DSC, Dice similarity coefficient; ICC, intraclass correlation coefficient; VB, vertebral bone; PM, psoas muscle; CM, core muscle; SAT, subcutaneous adipose tissue; sSAT, superficial SAT; dSAT, deep SAT; IPAT, intraperitoneal adipose tissue; RPAT, retroperitoneal adipose tissue; VAT, visceral adipose tissues

Our study addressed several limitations observed in previous research:

• We expanded the scope to include both abdominal and thigh BC components, covering a total of 15 tissue categories. This provided a more holistic understanding of BC distribution across multiple body regions.
• The proposed BioCompNet achieved state-of-the-art performance in BC quantification, with high DSC values and an average ICC exceeding 0.97, underscoring its reliability and precision across multiple body regions.
• Our study utilized 503 participants, offering a broad and diverse dataset that enhanced the robustness and generalizability of the findings. This diversity, paired with MRI imaging, ensures that our results were more widely applicable across different populations.

## Discussion

This study aimed to develop a robust tool for the rapid and accurate segmentation and quantification of abdominal and thigh BC features from MRI scans. The proposed BioCompNet could precisely segment up to 15 BC components, demonstrating high agreement between the automated results and physician assessments for volume measurements (ICC ≥ 0.881) and IMAT evaluation (*P*_trend_ < 0.001). The external test demonstrated the model’s excellent generalization across a heterogeneous population scanned with different scanning equipment and protocols. This automated workflow reduced physician imaging interpretation time per scan from 128.8 to just 0.12 min per scan. To our knowledge, this study represented the most extensive investigation of anatomical quantities in BC analysis using MRI. This comprehensive approach holds significant potential for facilitating further clinical research on the relationship between BC components and CMDs.

The proposed workflow enabled automated segmentation for 15 BC components across multiple anatomical regions, offering the most comprehensive quantitative tool available to date. Most previous models have primarily focused on the segmentation of adipose tissues [18,19,22,23,41,42], as illustrated in Table 5. However, growing research evidence emphasizes the significance of other BC components, such as muscles, IMATs, and bones, as imaging biomarkers for predicting mortality and major adverse cardiovascular events [26,42]. For instance, a previous study confirmed that a higher ratio of thigh SAT to abdominal adipose tissue was associated with a reduced risk of NAFLD [43]. In contrast to prior studies focused solely on the segmentation of isolated components, our model concurrently analyzed abdominal and thigh adipose tissues, muscles, and bones, supporting a more holistic analysis of whole-body composition and organ–tissue interaction.

Although tasked with segmenting multiple BC components concurrently, the proposed model achieved satisfactory segmentation accuracy due to high-quality manual annotation and network architecture design. While most existing DL models for the segmentation of BC have been developed based on CT scans, with reported DSCs exceeding 0.95 for VAT and SAT [18,19], MRI offers superior tissue contrast and is safer. However, MRI’s nonstandardized acquisition protocols and strong anisotropy pose additional challenges. Previous DL-based MRI studies have reported DSCs ranging from 0.85 to 0.975 for VAT and SAT [22,23,41]. To address these limitations, we leveraged the complementary characteristics of the fat and water sequences: Adipose tissues are more clearly visualized in the fat sequence, whereas muscles, bones, and vessels are better delineated in the water sequence [44]. During annotation, both sequences were jointly referenced to ensure high-quality GT labels. For model design, a dual-channel 2D U-Net architecture was employed to integrate both image sequences, enhancing tissue-specific segmentation while mitigating 3D anisotropy. Comparative experiments further confirmed the superior generalizability of the 2D approach over 3D architectures for this task. While BioCompNet performed slightly below the 2D nnU-Net on the internal test set, it consistently outperformed on the external test set. These results highlight the model’s robustness and generalization capability, supporting its suitability for large-scale population studies and real-world clinical applications.

Despite these strengths, this study identified that segmentation performance varied across different BMI groups, particularly for IPAT, RPAT, VAT, and vessels. The segmentation performance in the low-BMI group (<18.5) was statistically inferior, likely due to 2 primary factors: (a) Underrepresentation in the training dataset (only 7 cases, 1.4% of the total) and (b) the reduced volume and clarity of adipose tissue in individuals with a low BMI, which pose intrinsic challenges for model learning and annotation consistency. In addition, segmentation performance for VAT, IPAT, and RPAT in the abdominal region was statistically lower in the NAFLD group than in the non-NAFLD group. This suggests that hepatic fat-related pathophysiological changes may affect the imaging characteristics of surrounding adipose tissue, thereby influencing segmentation performance. These issues highlight the need for larger and more diverse cohorts with richer demographic and clinical features to ensure dataset balance and improve model generalizability. In addition, thigh vessel segmentation also exhibited statistically significant differences among several subgroups. This discrepancy is primarily due to the relatively small size and low pixel proportion of vascular structures compared to those of other components. Future iterations of the model will aim to expand the training dataset and explore architectural modifications, such as incorporating multiscale feature extraction or attention-based mechanisms, to enhance model sensitivity to subtle boundary cues and small structures.

Based on the accurate segmentation, the proposed workflow further enabled precise quantification of BC features. Features relevant to CMDs, such as the component areas and volumes of each component, are heavily reliant on accurate organ–tissue anatomical structures [35]. Previous studies commonly relied on manual or semiautomated 2D MRI [6,9], which were time-consuming, limited in scope, and susceptible to interreader variability. In contrast, our fully automated workflow addresses these limitations and extracts a broader range of features. The model demonstrated high accordance with physician measurements while drastically reducing processing time. Moreover, its compatibility with Digital Imaging and Communications in Medicine (DICOM) ensures seamless integration into existing hospital picture archiving communication systems (PACSs), enabling efficient processing of MRI scans in large-scale cohort studies and offering a clinically practical tool that does not increase the workload for physicians.

The proposed BioCompNet serves as both a research-enabling tool for biomarker discovery and a clinically applicable solution to support precision diagnostics and personalized interventions. As demonstrated in Fig. 6, substantial differences in BC distributions were observed across subgroups. Given the important roles of adipose tissue and skeletal muscle in metabolic regulation, these phenotypic differences may contribute to sex-specific or age-related susceptibility to metabolic disorders, thereby offering real-world evidence to support individualized preventive and therapeutic interventions [45,46]. Beyond research applications, automated quantification of BCs also holds great potential for informing clinical decision-making. Prior studies have demonstrated that specific BC phenotypes, such as increased VAT, elevated IMAT, or reduced skeletal muscle mass, are independently associated with insulin resistance, cardiovascular events, and all-cause mortality [4,6,26,42]. Additionally, the quantified BC-based distribution patterns like the thigh SAT/abdominal VAT ratio may offer a biomechanical insight related to energy dissipation efficiency or altered joint loading from sarcopenia, with potential applications in prosthetic design [16]. As such, the parameterized outputs of the proposed model can be directly integrated into structured radiology reports, providing accurate, reproducible, and interpretable metrics that support early identification of at-risk individuals and guide timely interventions. The demonstrated synergy between biological insights and engineering applications underscores our framework’s unique capacity to advance both clinical decision-making and clinically aided system development.

### Limitations

First, the BC components analyzed in this study were determined based on current evidence from in vivo, in vitro, and real-world studies on CMDs. Whether additional components have clinical significance remains to be further investigated. Second, although the postprocessing pipeline was fully automated, visually identified segmentation errors were manually corrected using the ITK-SNAP or 3D Slicer platform before quantification to ensure the reliability of final results. In future applications, a user-friendly interactive interface should be developed to allow clinicians to review and adjust outputs as needed. Third, enhancing the model’s generalizability across datasets with diverse characteristics will require a larger, more balanced training and testing cohort drawn from multicenter and heterogeneous populations, along with further architectural optimization. Lastly, the clinical implications of these imaging-derived BC features, particularly their associations with CMD risk, have not been fully elucidated. Large-scale, multicenter cohort studies are essential to validate these associations and establish the model’s utility in supporting clinical decision-making, including risk stratification, diagnosis, and treatment planning.

## Conclusion

This study developed a fully automated workflow for segmenting abdominal and thigh MRI scans to rapidly quantify BC components using DL. The workflow offers the most extensive quantitative analysis of BC components for multiple anatomical regions, achieving performance comparable to or surpassing that of previously reported DL-based models. The proposed BioCompNet demonstrated high consistency in quantification accuracy compared to physicians and great generalization to external test datasets, thus addressing an essential requirement for developing clinically practical tools aimed at exploring the association between BC components and diseases. In addition, the modular design of the proposed workflow enables seamless integration into hospital PACSs, providing fast, reproducible outputs to support clinical decision-making without increasing workload. Future studies will require large-scale, multi-site, and multi-device cohort investigations to further optimize the model’s performance. We believe that this study and others will help build momentum to advance research in this field, prompting further consideration of whether MRI-based BC components analysis should be utilized as an indicator to proactively investigate and manage populations at risk or affected by CMDs.

## Data Availability

The datasets supporting this study’s findings are restricted under China’s Human Genetic Resources Administration Regulations (State Council Order No. 717). Researchers may submit data access requests via email to corresponding author Prof. Weiping Jia, who will respond within 10 business days. Upon initial agreement, the data requester, in conjunction with the corresponding author, must submit a joint application for data sharing to the Ministry of Science and Technology of China (MOST). Data transfer will be executed only after obtaining official MOST authorization.
