# Supplementary material for: BioCompNet: A Deep Learning Workflow Enabling Automated Body Composition Analysis toward Precision Management of Cardiometabolic Disorders
Source: Cyborg Bionic Syst. 2025 Aug 20;6:0381. doi: 10.34133/cbsystems.0381 (PMC12367250; doi:10.34133/cbsystems.0381)
Supplement: Supplementary 1 — Tables S1 and S2 Figs. S1 to S4 [file cbsystems.0381.f1.docx]

**Supplementary Materials**

**Table S1. MR Imaging Examination Acquisition Parameters.**

| **Parameters** | **Model development and internal test set** | **External validation set** |
| --- | --- | --- |
|  | **Abdomen/ Thigh** | **Abdomen/ Thigh** |
| Manufacturer | GE Signa HDxt;  GE Medical Systems | SIEMENS Prisma;  UIH uMR 780 |
| MR imaging sequence | 3-pl Loc/WATER: BH Ax; LAVA-Flex Mask/FAT: BH Ax LAVA-Flex Mask | T1_vibe_dixon_tra_p4_bh_F;  T1_vibe_dixon_tra_p4_bh_W/  Gre_quick_wfi |
| Sequence type | GR | GR |
| Sequence Variant | SS\SK | SP\OSP |
| Acquisition type | 3D | 3D / 2D |
| Echo time (msec) | 2.016/1.674 | 1.29 / 82 |
| Acquisition time (msec) | 193145/193244 | 130043 / 161946 |
| Coil type | BODY | BODY |
| Flip Angle | 12 | 9 / 12 |
| Slice thickness (mm) | 10 | 5 (min-max: 3-6) |
| Image pixel spacing | 0.8203 | 1.1875 |
| Image size (mm) | 512 x 512 | 320 x 260 / 294 x 448; 320 x 320 |
| Images in acquisition | 8 | 64 / 24 |

**Table S2. Average segmentation DSC for the ablation study across different model variants.**

| **Component** | **Fat-only**  **with DA** | **Water-only**  **with DA** | **Dual-sequence without DA** | **Dual-sequence**  **with DA** | |
| --- | --- | --- | --- | --- | --- |
| **Internal test set** | | | | |  |
| **Abdominal** |  |  |  |  | |
| VB | 0.947 ± 0.033 | 0.952 ± 0.025 | 0.958 ± 0.022 | 0.962 ± 0.021 | |
| PM | 0.958 ± 0.034 | 0.961 ± 0.036 | 0.964 ± 0.034 | 0.970 ± 0.021 | |
| CM | 0.949 ± 0.019 | 0.952 ± 0.021 | 0.956 ± 0.027 | 0.960 ± 0.018 | |
| SAT | 0.968 ± 0.022 | 0.973 ± 0.016 | 0.974 ± 0.014 | 0.976 ± 0.011 | |
| dSAT | 0.916 ± 0.035 | 0.920 ± 0.031 | 0.920 ± 0.032 | 0.945 ± 0.023 | |
| sSAT | 0.922 ± 0.034 | 0.936 ± 0.038 | 0.938 ± 0.027 | 0.926 ± 0.030 | |
| VAT | 0.958 ± 0.025 | 0.961 ± 0.030 | 0.966 ± 0.021 | 0.968 ± 0.019 | |
| IPAT | 0.894 ± 0.049 | 0.901 ± 0.046 | 0.921 ± 0.036 | 0.926 ± 0.033 | |
| RPAT | 0.819 ± 0.074 | 0.821 ± 0.061 | 0.857 ± 0.053 | 0.866 ± 0.049 | |
| Average | 0.926 ± 0.028 | 0.931 ± 0.026 | 0.939 ± 0.023 | 0.944 ± 0.019 | |
| **Thigh** |  |  |  |  | |
| Femur | 0.982 ± 0.007 | 0.984 ± 0.005 | 0.989 ± 0.003 | 0.990 ± 0.004 | |
| Vessel | 0.867 ± 0.063 | 0.865 ± 0.049 | 0.870 ± 0.058 | 0.877 ± 0.052 | |
| SAT | 0.981 ± 0.004 | 0.981 ± 0.006 | 0.980 ± 0.008 | 0.982 ± 0.007 | |
| Muscle | 0.984 ± 0.006 | 0.986 ± 0.003 | 0.994 ± 0.002 | 0.994 ± 0.002 | |
| Average | 0.954 ± 0.018 | 0.954 ± 0.014 | 0.940 ± 0.131 | 0.961 ± 0.015 | |
| **External test set** | | | | |  |
| **Abdominal** |  |  |  |  | |
| VB | 0.942 ± 0.035 | 0.956 ± 0.019 | 0.894 ± 0.179 | 0.960 ± 0.023 | |
| PM | 0.932 ± 0.084 | 0.933 ± 0.054 | 0.929 ± 0.142 | 0.966 ± 0.047 | |
| CM | 0.930 ± 0.067 | 0.942 ± 0.041 | 0.957 ± 0.036 | 0.968 ± 0.026 | |
| SAT | 0.950 ± 0.016 | 0.945 ± 0.089 | 0.968 ± 0.060 | 0.985 ± 0.008 | |
| dSAT | 0.891 ± 0.075 | 0.889 ± 0.075 | 0.892 ± 0.081 | 0.916 ± 0.056 | |
| sSAT | 0.907 ± 0.046 | 0.916 ± 0.048 | 0.910 ± 0.077 | 0.939 ± 0.031 | |
| VAT | 0.921 ± 0.035 | 0.925 ± 0.052 | 0.939 ± 0.086 | 0.963 ± 0.058 | |
| IPAT | 0.860 ± 0.072 | 0.861 ± 0.074 | 0.872 ± 0.104 | 0.906 ± 0.066 | |
| RPAT | 0.812 ± 0.163 | 0.814 ± 0.158 | 0.803 ± 0.118 | 0.836 ± 0.116 | |
| Average | 0.905 ± 0.054 | 0.909 ± 0.058 | 0.907 ± 0.091 | 0.938 ± 0.042 | |
| **Thigh** |  |  |  |  | |
| Femur | 0.932 ± 0.041 | 0.915 ± 0.052 | 0.957 ± 0.028 | 0.960 ± 0.024 | |
| Vessel | 0.833 ± 0.054 | 0.836 ± 0.029 | 0.815 ± 0.045 | 0.841 ± 0.035 | |
| SAT | 0.922 ± 0.094 | 0.934 ± 0.075 | 0.957 ± 0.012 | 0.961 ± 0.012 | |
| Muscle | 0.931 ± 0.060 | 0.923 ± 0.052 | 0.981 ± 0.006 | 0.984 ± 0.004 | |
| Average | 0.905 ± 0.048 | 0.902 ± 0.038 | 0.928 ± 0.017 | 0.936 ± 0.047 | |

Note. — Data are reported as mean ± standard deviation. DSC, Dice similarity coefficients; DA, data augmentation; VB, vertebral bone; PM, psoas muscles; CM, core muscles; SAT, subcutaneous adipose tissues; VAT, visceral adipose tissues.; IPAT, intraperitoneal adipose tissue; RPAT, retroperitoneal adipose tissue.

# Fig. S1. Manual annotated labels in abdominal and thigh.

# Fig. S2. Annotation workflow for all datasets in model development.


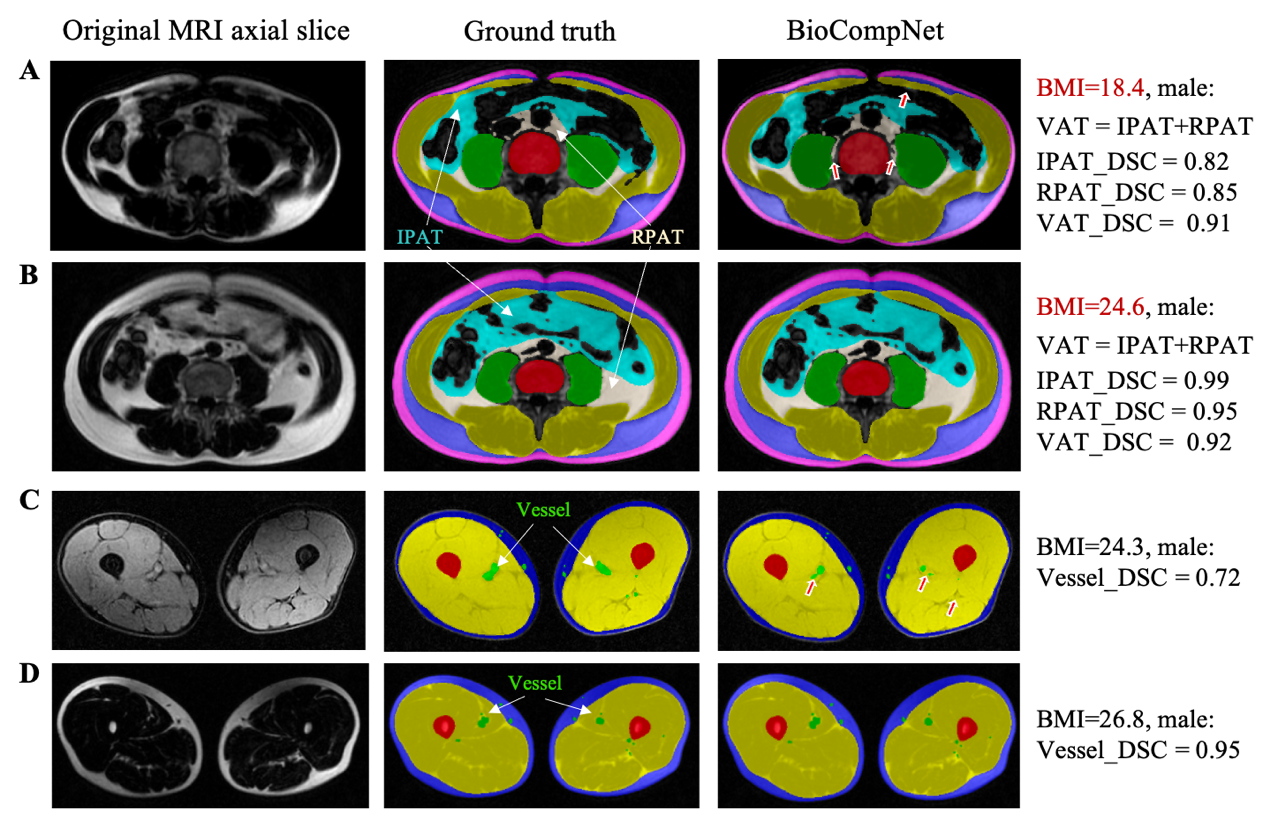


**Fig. S3. Segmentation results of abdominal and thigh body composition components using BioCompNet across four cases with varying BMI.**
(a) and (b) show segmentation results of VAT, IPAT, RPAT in two cases with different BMI levels. It can be observed that in the lower-BMI case, both IPAT and RPAT are less abundant and more challenging to segment accurately. (c) and (d) illustrate segmentation results of thigh blood vessels in two cases with similar BMI. Despite the comparable BMI values, significant differences in vessel segmentation performance are evident. This variation is likely due to the small relative size of blood vessels in the overall segmentation target, making them more susceptible to segmentation inconsistencies.

DSC, Dice similarity coefficients; BMI, body mass index; MRI, magnetic resonance imaging; VAT, visceral adipose tissue; IPAT, intraperitoneal adipose tissue; RPAT, retroperitoneal adipose tissue.


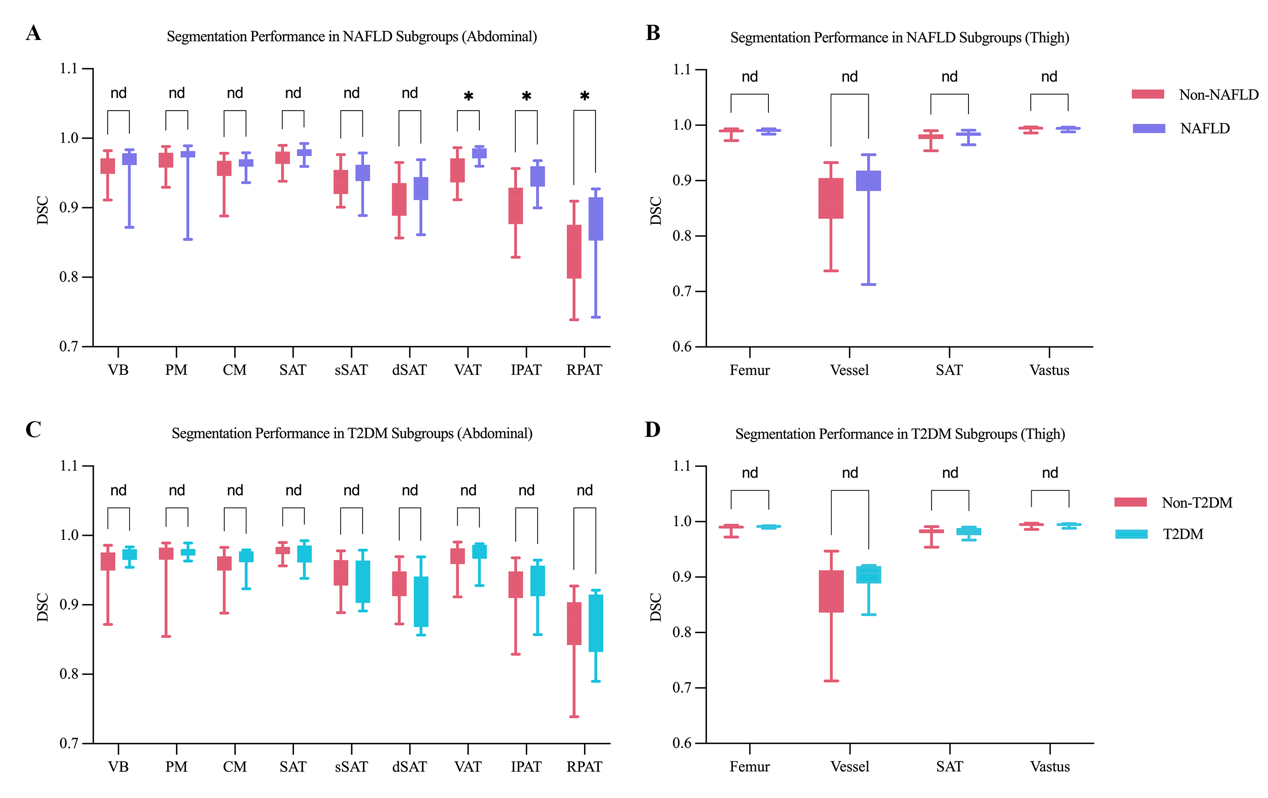


**Fig. S4. Subgroup analysis of segmentation performance (measured by DSC) in disease versus normal groups.** (a–b) Comparison of segmentation performance between NAFLD and normal participants for abdominal (a) and thigh (b) compartments. Statistically significant differences were observed in the segmentation of VAT, IPAT, and RPAT between NAFLD and Non-NAFLD groups (indicated by *), while other compartments showed no significant difference (nd). (c–d) Comparison of segmentation performance between T2DM and Non-T2DM participants for abdominal (c) and thigh (d) compartments. No statistically significant differences were observed between groups across all compartments (nd). Statistical comparison was performed using independent-sample t-tests. Boxes represent interquartile range (IQR), whiskers indicate 1.5 × IQR, and center lines denote the median.

DSC, Dice similarity coefficient; VB, vertebral bone; PM, psoas muscles; CM, core muscles; SAT, subcutaneous adipose tissues; sSAT, superficial subcutaneous adipose tissues; dSAT, deep subcutaneous adipose tissues; VAT, visceral adipose tissues; IPAT, intraperitoneal adipose tissue; RPAT, retroperitoneal adipose tissue; NAFLD, non-alcoholic fatty liver disease; T2DM, type 2 diabetes mellitus; nd, no statistically significant differences.
